# Use of PEGylated Recombinant Human Growth Hormone in Chinese Children with Growth Hormone Deficiency: A 24-Month Follow-Up Study

**DOI:** 10.1155/2019/1438723

**Published:** 2019-09-19

**Authors:** Yu Qiao, Zengmin Wang, Jinyan Han, Guimei Li

**Affiliations:** Department of Pediatrics, Shandong Provincial Hospital Affiliated to Shandong University, 9677 Jingshi Road, Jinan 250021, Shandong, China

## Abstract

**Objective:**

Once-weekly PEGylated recombinant human growth hormone (rhGH) is the sole long-acting GH formulation available currently for pediatric patients with GH deficiency (GHD). The aim of this study was to evaluate the efficacy and safety of PEGylated rhGH therapy compared to daily rhGH therapy in GHD children treated for two years.

**Methods:**

A total of 98 children (49 children for the PEGylated rhGH group and 49 children for the daily rhGH group) with GHD were enrolled in this single-center, prospective, nonrandomized cohort study. PEGylated rhGH or daily rhGH was administered for 2 years. Height, height SDS, height velocity (HV), IGF-1, bone age (BA), and adverse events were determined throughout the treatment.

**Results:**

HV significantly increased over the baseline and was similar in both groups. In the PEGylated rhGH cohort, the mean ± SD HV was improved from 3.78 ± 0.78 cm/y at the baseline to 12.44 ± 3.80 cm/y at month 3, to 11.50 ± 3.01 cm/y at month 6, to 11.00 ± 2.32 cm/y at month 12, and finally 10.08 ± 2.12 cm/y at month 24 in the PEGylated rhGH group. In the daily rhGH group, HV was 3.36 ± 1.00 cm/y at baseline, increasing to 12.56 ± 3.71 cm/y at month 3, to 11.82 ± 2.63 cm/y at month 6, to 10.46 ± 1.78 cm/y at month 12, and to 9.28 ± 1.22 cm/y at month 24. No serious adverse event related to PEGylated rhGH or daily rhGH occurred during the 24-month study.

**Conclusion:**

PEGylated rhGH replacement therapy is effective and safe in pediatric patients with GHD. The adherence to once-weekly PEGylated rhGH therapy is superior to daily rhGH in children with GHD.

## 1. Introduction

Pediatric patients with growth hormone deficiency (GHD) entail years of injections with recombinant human growth hormone (rhGH). As the rhGH's serum half-life is short, only 3.4 hours after subcutaneous (SC) injection and 0.36 hours after intravenous (IV) injection [1], the most widely used regimen is daily SC injections, which can be distressing and inconvenient for some patients and can lead to poor compliance and dissatisfying treatment outcomes.

In teenagers, approximately 23% omit two or more injections per week [2]. Several long-acting formulations via different pharmacological strategies (e.g., sustained-release preparations, prolonged half-life derivatives, and new injectors such as electronic injection pen) have been studied in pediatric GHD patients, with the hope of improved compliance, and without adverse effects [3]. PEGylation, a pharmacological technology to prolong the serum half-life of therapeutic proteins through covalent modification of proteins with polyethylene glycol (PEG), is deemed as one of the most successful techniques to increase solubility and physical and chemical stability and in concert with avoidance of toxicity and immunogenicity [4].

In China, once-weekly PEGylated rhGH is the sole long-acting GH formulation available currently for pediatric patients with GHD. By conjugating a branched PEG molecule to amino groups of rhGH, thereby increasing the hydrodynamic size of GH and reducing renal clearance, the circulating hormone has a prolonged duration of action. Phase II and III multicenter, randomized studies from 6 hospitals in China confirmed that PEG-rhGH at a dose of 0.2 mg/kg/week is effective and safe for children with GHD during 25 week treatment [5]. In the phase II and III study, significantly greater improvement in the height standard deviation scores was associated with PEG-rhGH through the treatment. In summary, we evaluated the longer-term efficacy and safety of PEGylated rhGH for two-year treatment of GH-deficient children.

## 2. Materials and Methods

### 2.1. Patients

The eligible patients (61 males and 37 females, age from 3 to 13 years) were prepubertal (Tanner stage 1), with the diagnosis of GHD as determined by the following inclusion criteria: (1) short stature with height standard deviation score (HtSDS) <−2 based on the Chinese general population standard for age or <3rd percentile for chronological age (CA) or height velocity (HV) <5 cm/year; (2) peak GH concentrations less than 10 ng/dl in response to two pharmacological agents on two separate days (arginine 0.5 g/kg, maximum dose of 30 g, and levodopa 10 mg/kg, maximum dose of 500 mg); (3) bone age (BA) less than 10 years for girls and 12 years for boys, with a minimum of 1 year delay compared to the CA. All patients (*n* = 98) were diagnosed with isolated GHD. Hypothalamic-pituitary magnetic resonance imaging (MRI) was performed to exclude masses or congenital malformations. Prior to GH treatment, written informed consent from a parent or legal guardian was signed. Patients were excluded if they had growth failure related to other causes, such as diabetes mellitus, impaired fasting glucose, tumors, congenital skeletal abnormalities, congenital heart disease, chronic illness, confirmed diagnosis of an eponymous syndrome (e.g., Turners, Noonan, Prader-Willi, and Russell Sliver), or poorly controlled MPHD. Every patient received relevant extensive education and training on the use and basic pharmacokinetics of rhGH before the initiation of rhGH treatment.

### 2.2. Treatments

49 patients received PEGylated rhGH (GeneScience Pharmaceuticals, Changchun, People's Republic of China) at a once-weekly dose of 0.2 mg/kg/week. In contrast, 49 patients received daily SC. rhGH injection (Jintropin AQ, GeneScience Pharmaceuticals) at a dose of 0.30 mg/kg/w. These two kinds of rhGH were self-paid by the parents. No insurance coverage was available for these children. The treatment and follow-up were performed in Shandong Provincial Hospital Affiliated to Shandong University between May 1, 2012 and June 30, 2018. The protocol of this study was approved by the Ethics Committee of Shandong Provincial Hospital (Jinan, China).

### 2.3. Study Measurements

All patients were assessed at the baseline and 3, 6, 12, and 24 months after initiation of treatment. At baseline and each interval, height and weight were measured by the same auxologist. HV, HtSDS, and body mass index (BMI) were calculated. Blood samples were obtained for insulin-like growth factor 1 (IGF-1), thyroid function (free thyroxine FT4, free triiodothyronine FT3, and thyroid-stimulating hormone TSH), adrenocorticotropic hormone (ACTH), cortisol, glucose metabolism (glycated hemoglobin HbA1c and fasting blood glucose), renal function (urea nitrogen BUN, and creatinine), and complete blood count. All these blood samples were obtained after an overnight fast. Serum concentration of GH and IGF-1 were measured by using chemiluminescence assay (Immulite 2000; Siemens Health Care Diagnostics, USA). Intra-assay and interassay coefficients of variation (CV) declared by the manufacturer were 2.5% and 7.5%, respectively, for IGF-1 measurement. Intra-assay and interassay CVs for GH concentration are 4.5% and 5.8%, respectively. BA radiography was determined by the TW3 method [6]. In addition, injection-site reactions and tolerability were monitored to assess the possible side effects of GH treatment. All of the children completed hypothalamic-pituitary MRI, which was used by a 3.0 T magnetic resonance scanning machine (Siemens & Co, Germany). Pituitary height was measured and compared with normal values for the corresponding age [7]. We transformed IGF-1 into IGF-1 standard deviation scores based on normative values from a normal population [8].

The data are presented as mean ± SD, and 95% confidence intervals were used where indicated. The changes in HV, HtSDS, BA, IGF SDS [8], and blood measurements were compared using the paired *t* test. Independent *t* tests were used to assess indexes between the two groups. Multiple regression analysis was applied to analyze the relationship between 12-month HV and the baseline characteristics.

## 3. Results

### 3.1. Patients

A total of 100 patients were enrolled (PEGylated rhGH group, *n* = 49 or daily rhGH group, *n* = 49) and received GH treatment. The study was not randomized: parents often decided which GH therapy they preferred, and notably, the PEGylated rhGH preparation is more expensive. All patients accomplished 12 months of treatment (PEGylated rhGH group, *n* = 49, vs daily rhGH group, *n* = 49), and 85 (87%) patients completed 24 months (PEGylated rhGH group, *n* = 38, vs daily rhGH group, *n* = 47). In the PEGylated group, all 38 patients were with PEGylated rhGH throughout. Five patients of the PEGylated rhGH group switched to using daily rhGH in view of the high price of PEGylated rhGH. Two patients were lost to follow-up after the 12-month visit due to concern on safety of rhGH. Four patients were still in follow-up that did not reach the 24-month milestone. In the daily rhGH group, 2 patients were out of touch after 12 months of daily rhGH treatment.

### 3.2. Baseline Characteristics

The baseline characteristics of the enrolled patients are summarized in Table 1. There was no statistically significant difference between groups for age, height, IGF-1 SDS, and BMI at entry. All of the patients were preadolescent, and bone age (BA)/chronological age (CA) indicated retardation of bone maturation.

### 3.3. Efficacy

#### 3.3.1. Height Velocity

**Figure 1:**
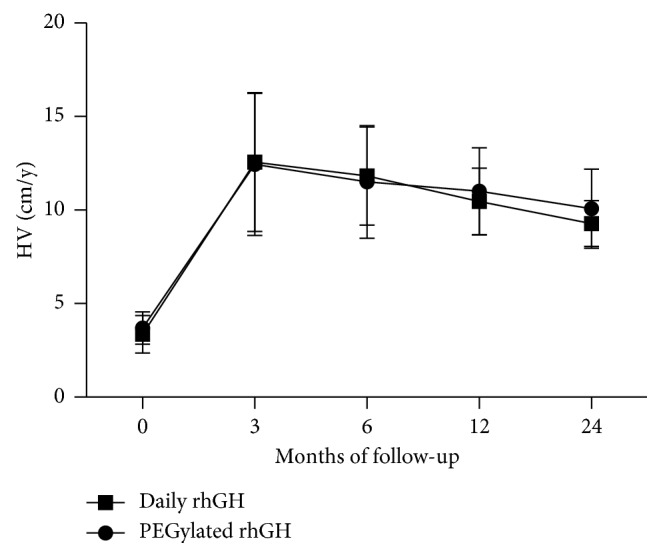
Temporal trend in HV over time in GHD children treated with PEGyated rhGH (weekly) or daily rhGH (daily) for 24 months. Values represent mean ± SD.

Annualized HV in both groups at month 3, month 6, month 12, and month 24 improved. In PEGylated rhGH group, annualized HV increased from 3.78 ± 0.78 cm/y (*n* = 49) at the baseline to 12.44 ± 3.80 cm/y (*n* = 49) at month 3, to 11.50 ± 3.01 cm/y (*n* = 49) at month 6, to 10.57 ± 2.06 cm/y (*n* = 49) at month 12, and to 10.08 ± 2.12 cm/y (*n* = 38) at month 24, finally. A near-identical trend was observed in the daily rhGH group: annualized HV increased from 3.36 ± 1.00 cm/y (*n* = 49) at the baseline to 12.56 ± 3.71 cm/y (*n* = 49) at month 3, to 11.82 ± 2.63 cm/y (*n* = 49) at month 6, to 10.46 ± 1.78 cm/y (*n* = 49) at month 12, and to 9.28 ± 1.22 cm/y (*n* = 47) at month 24 (Figure 1). As the height velocity did not statistically differ at each visit benchmark (*p*=0.877 at month 3, *p*=0.580 at month 6, *p*=0.200 at month 12, and *p*=0.055 at month 24), data of annualized HV were aggregated to analyze the trend. The mean annualized HV was from 3.53 ± 0.95 cm/y (*n* = 98) at the baseline to 12.50 ± 3.74 cm/y (*n* = 98) at month 3, to 11.66 ± 2.82 cm/y (*n* = 98) at month 6, to 10.72 ± 2.06 cm/y (*n* = 98) at month 12, and then to 9.56 ± 1.62 cm/y (*n* = 85) at month 24, finally. As anticipated, annualized HV decreased progressively with time.

**Figure 2:**
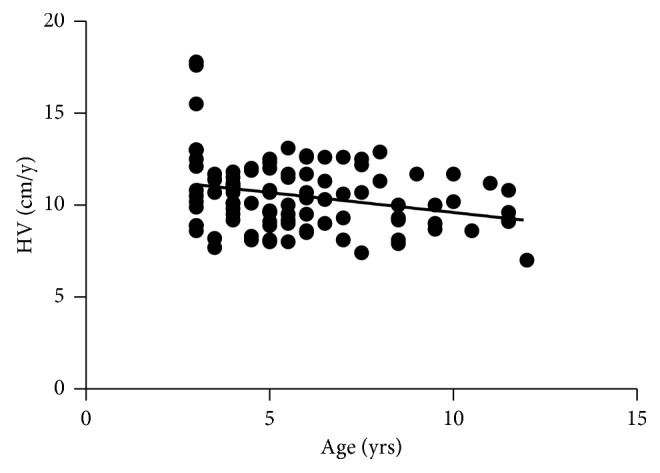
Relationship of 12-month HV to age at initiation of treatment in patients with GHD (*r* = −0.22, *p*=0.0065).

**Figure 3:**
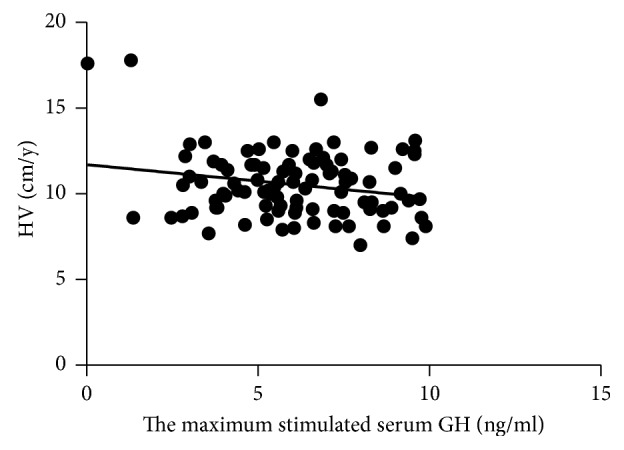
Relationship of 12-month HV to the maximum stimulated serum GH in patients with GHD (*r* = −0.19, *p*=0.0368).

Discrete variables for univariate analysis showed no significant relationship between 12-month HV and sex. Moreover, analysis of continuous baseline characteristics found that pretherapy HV, BA, BMI, and height of hypophysis had no relationship with 12-month HV in the multiple regression analysis separately (*p* values were 0.1825, 0.0069, 0.2942, and 0.3104, respectively). HV depended on age and the maximum stimulated serum GH concentration negatively (*p* values were 0.0065 and 0.0368, respectively) (Figures 2 and 3).

**Figure 4:**
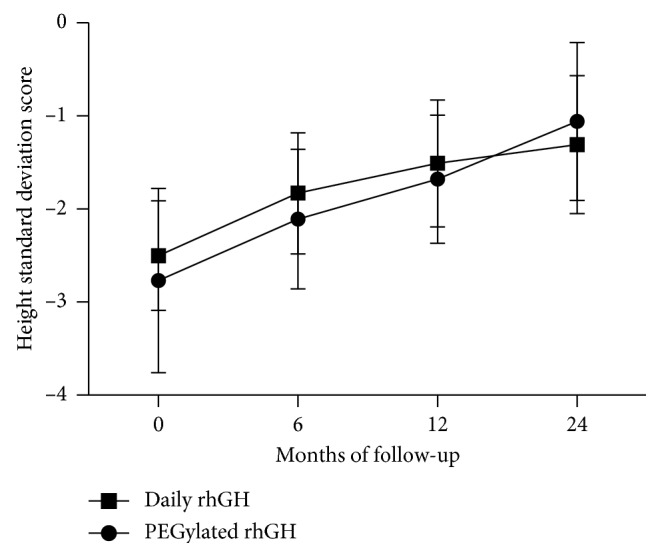
Height SDS over time in GHD children treated with PEGyated rhGH (weekly) or daily rhGH (daily) for 24 months. Values represent mean ± SD.

During the period of PEGylated rhGH or daily rhGH treatment, HtSDS increased gradually in all patients with the passage of treatment time. The respective HtSDS values for the PEGylated rhGH vs daily rhGH cohorts were −2.57 ± 0.75 vs −2.50 ± 0.59 (*p*=0.099) at the baseline, increasing to −2.11 ± 0.75 vs −1.83 ± 0.65 (*p*=0.054) at 6 months, to −1.68 ± 0.69 vs −1.51 ± 0.68 (*p*=0.232) at 12 months, and to −1.06 ± 0.85 vs −1.13 ± 0.74 (*p*=0.738) at 24 months (Figure 4). Notably, at each visit milestone, there was no statistical difference between the two treatment groups. The pattern of change in mean HtSDS was consistent with catch-up growth. The mean change in HtSDS was significant from the baseline to 24 months between these two groups (*p* ≤ 0.001). There was no difference in bone advancement between the two groups following 24 months of treatment.

#### 3.3.2. IGF-1

**Figure 5:**
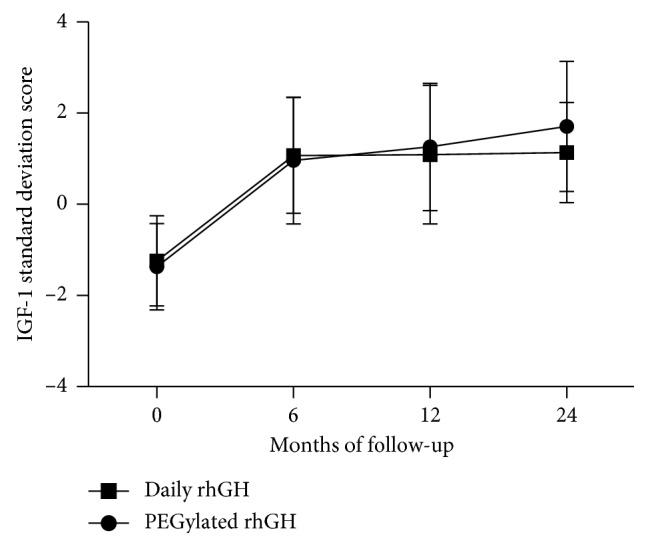
IGF SDS over time in GHD children treated with PEGyated rhGH (weekly) or daily rhGH (daily) for 24 months. Values represent mean ± SD.

The mean value of IGF-1 SDS based on chronological age at the baseline was −1.28 ± 0.98 for the PEGylated rhGH group and −1.24 ± 0.99 in the daily rhGH group (*p*=0.491), and respective means were 0.96 ± 1.39 and 1.07 ± 1.27 at month 6 (*p*=0.651), 1.26 ± 1.40 and 1.09 ± 1.52 at month 12 (*p*=0.559), and 1.71 ± 1.43 and 1.14 ± 1.10 at month 24 (*p*=0.074), with no statistical significance between the treatment groups at month 6, month 12, or month 24 (Figure 5).

#### 3.3.3. Bone Age

Mean change (SD) from baseline to 12 months in bone age was 1.08 ± 0.48 years in the PEGylated rhGH group (*n* = 49) and 1.11 ± 0.49 years in the daily rhGH group (*n* = 49). There was no statistical difference in these two groups (*p*=0.92). The increased BA from the baseline to 24 months was 1.96 ± 0.73 years in the PEGylated rhGH group (*n* = 38) and 2.27 ± 0.93 years in the daily rhGH group (*n* = 47). There was also no statistical difference of BA during the two-year treatment (*p*=0.103).

### 3.4. Safety

Adverse events in the PEGylated group were the same as those in the daily rhGH group. There have been no confirmed cases of type 1 or type 2 diabetes mellitus with either rhGH treatment. The glucose level of PEGylated rhGH group was 4.75 ± 0.69 mmol/L at the baseline, 5.14 ± 0.43 mmol/L at month 12, and 5.13 ± 0.52 mmol/L at month 24. For the daily rhGH group, the glucose level was 4.84 ± 0.54 mmol/L at the baseline, 5.29 ± 0.43 mmol/L at month 12, and 5.26 ± 0.43 mmol/L at month 24. There was no significant difference during the treatment of PEGylated rhGH at month 12 (*p*=0.106) or month 24 (*p*=0.310) so as the change of HbA1c (*p*=0.310 at month 12, *p*=0.888 at month 24). Injection-site lipoatrophy was not encountered in either group. No significant peripheral edema, headache, or injection-site reactions were noted. No severe adverse event was detected during the treatment between these two groups. Of note, 2 patients in the PEGylated group developed hypothyroidism vs 3 patients in the daily rhGH group. Administration of low dose L-thyroxin has normalized thyroid function. All of the side effects in both groups of patients are illustrated in the Table 2.

## 4. Discussion

In order to increase adherence to growth hormone therapy in the previous study, several formulations have been developed. PEGylation reduces renal clearance. After the discontinuation of early PEGylated rhGH named PHA-794438 and NNC126-0083, Jintrolong® is the only commercially available PEGylated rhGH currently [3]. The results of our analysis demonstrated that Jintrolong® was effective, well-tolerated, and convenient in Chinese children with growth deficiency.

There was no difference in augmentation of HV between the PEGylated rhGH group and the daily rhGH group at each visiting time. As previously reported, HV was not related to sex, BA, pretherapy HV, height of hypophysis, and BMI. The growth rate is negatively correlated with age and peak GH levels. Replacement of rhGH therapy in GHD children at an early age is more likely to attain catch-up growth and a normalization of adult height [9]. GHD children will improve their chances of achieving their genetic height potential because of early rhGH therapy [10]. During the first year treatment in these two groups, the BA was both advanced nearly one year, indicating no undue advancement of skeletal maturation. Bone maturation and height progression were also parallel during the two-year treatment.

An early PEGylated rhGH preparation named NNC126-0083 was due to the unsatisfactory once-weekly IGF-1 profile [11]. Using Jintrolong® as a new kind of PEGylated rhGH, IGF-1 level increased steadily [12]. Our study demonstrated that the concentration of IGF-1 in both PEGylated rhGH group and daily rhGH group reached the upper normal range during the first 6 months. Some guidelines also recommended the level of IGF-1 to adjust the dose of rhGH [13]. Serum IGF-1 level can also assess the adherence to rhGH injections [14]. It remains contestable whether high serum IGF-1 concentrations result in better height outcome or long-term risks [15].

Our follow-up investigation also showed that a weekly PEGylated rhGH had better safety than a daily rhGH. For example, values of fasting glucose and glycosylated hemoglobin remained unchanged from the baseline to 12 months and 24 months in the PEGylated rhGH group and the daily rhGH group. No patients developed diabetes. Several studies have reported a link between the development of diabetes and rhGH treatment [16]. GH may impact glucose homeostasis through a negative direct and indirect effect on the sensitivity of insulin. However, other studies have not established the same relationship. Baronio et al. conducted a median period of 6-year surveys of 99 GHD children and no deterioration in glucose homeostasis was found [17]. In terms of glucose homeostasis, this study confirmed the safety of GH treatment in GHD children and affirmed that regular glucose tolerance tests were unnecessary.

In our trial, the common adverse events were transient, mild, and consistent with safety events reported in the labels for rhGH products. Mild, easily treated hypothyroidism was found in 5 patients (3 in PEGylated group and 2 in daily group). In all these 5 patients in both groups, we observed a decrease in the serum FT4 level and no changes in the serum TSH level after rhGH administration. We inclined to the type of central hypothyroidism. Different mechanisms have been suggested to explain the relationship between GH and thyroid function. One mechanism demonstrated an inhibition of TSH release via an increased somatostatinergic tone or by a T3 negative feedback mechanism within the thyrotropes due to increase in T3 production from T4 deiodination at central level. The other mechanism suggests an increase in extrathyroidal conversion of T4 to triiodothyronine (T3) chiefly mediated directly by GH, or through IGF-1. This also can be found by reducing reverse-T3 (rT3) and/or increasing T3/T4 ratio during rhGH therapy at the peripheral level [18–20].

As for long-term safety, a cohort comprising 23,984 patients treated with rhGH in 8 European countries since introduction in 1989 did not find whether rhGH therapy affects the risk of cancer incidence or mortality [21]. The latest data from GeNeSIS observational program also found no increase in risk of mortality comparing rhGH-treated children with children in the general population [22].

In the study, none of the patients reported injection-site lipoatrophy after 24-month PEGylated rhGH injection. A previous study reported that a total of 13 cases of injection-site lipoatrophy among 105 subjects with GHD treated with PEGylated rhGH named PHA-794428 [23]. All lipoatrophy lesions in these cases resolved in 8–12 weeks. Acquired lipoatrophy is generally localized and refers to a limited, well-circumscribed subcutaneous depression matching an area of fat loss. Long-acting rhGH with different pharmacokinetic and pharmacodynamic profiles compared to daily rhGH should continue surveillance during and in the years after treatment and even in old age in those who continue therapy [24].

In conclusion, PEGylated rhGH will play a more important role in GHD children for long-term rhGH replacement. It will increase convenience and compliance in GHD children without increasing safety concerns. Meanwhile, improvements of PEGylated rhGH in the therapeutic aspect were similar to that of daily rhGH. Weekly injection will reduce the needle-related fear of children. The early treatment of rhGH was proven to improve HV, final adult height, and psychosocial development of pediatric patients. The long-term efficacy of PEGylated rhGH in children with GHD needs to be explored further to assess its potential superiority.

## Figures and Tables

**Table 1:** Demographic and baseline characteristics.

Characteristics	Group		
	PEGylated rhGH	Daily rhGH	*p* value
Number of patients (*n*)	49	49	
Male	26	33	
Female	23	16	
Chronological age (yrs)	5.41 ± 2.37	6.25 ± 2.42	0.373
Bone age (yrs)	4.47 ± 2.29	4.37 ± 2.47	0.237
BA/CA	0.66 ± 0.26	0.69 ± 0.29	0.198
Previous height velocity (cm/yr)	3.78 ± 0.78	3.36 ± 1.00	0.091
Height SDS	−2.57 ± 0.75	−2.50 ± 0.59	0.490
Parental height (cm)			
Father height	169 ± 5.40	169 ± 5.21	0.674
Mother height	158 ± 4.90	156 ± 4.42	0.851
BMI (kg/m^2^)	15.27 ± 1.45	15.13 ± 1.00	0.417
IGF-1 SDS	−1.28 ± 0.98	−1.24 ± 0.99	0.750
Peak GH (ng/ml)	5.91 ± 2.34	6.21 ± 2.02	0.202
Height of hypophysis (mm)	3.58 ± 0.74	3.89 ± 1.14	0.340

Data is represented as mean ± SD.

**Table 2:** Treatment-related adverse events reported in each group.

	PEGylated rhGH group (*n* = 49)	Daily rhGH group (*n* = 49)	
	*N* (%)	*N* (%)	
Hypothyroidism	2 (4.1%)	3 (6.1%)	*p*=1.00
Peripheral edema	0	0	
Headache	0	0	
Injection-site lipoatrophy	0	0	
Diabetes mellitus	0	0	

## Data Availability

The data used to support the findings of this study are included within the article. All of the data in this study were collected by using query system of medical record of Shandong Provincial Hospital Affiliated to Shandong University and by enquiring the parents of GHD children in clinic.
